# Tunable Photoresponse in a Two-Dimensional Superconducting Heterostructure

**DOI:** 10.3390/nano13030421

**Published:** 2023-01-19

**Authors:** Zijie Ji, Ruan Zhang, Shuangxing Zhu, Feifan Gu, Yunmin Jin, Binghe Xie, Jiaxin Wu, Xinghan Cai

**Affiliations:** 1 National Key Laboratory of Science and Technology on Micro/Nano Fabrication, Shanghai Jiao Tong University, Shanghai 200240, China; 2 Department of Micro/Nano Electronics, School of Electronic Information and Electrical Engineering, Shanghai Jiao Tong University, Shanghai 200240, China

**Keywords:** niobium diselenide, van der Waals heterostructure, superconducting photodetector, gate modulation

## Abstract

The photo-induced superconducting phase transition is widely used in probing the physical properties of correlated electronic systems and to realize broadband photodetection with extremely high responsivity. However, such photoresponse is usually insensitive to electrostatic doping due to the high carrier density of the superconductor, restricting its applications in tunable optoelectronic devices. In this work, we demonstrate the gate voltage modulation to the photoresponsivity in a two-dimensional NbSe_2_-graphene heterojunction. The superconducting critical current of the NbSe_2_ relies on the gate-dependent hot carrier generation in graphene via the Joule heating effect, leading to the observed shift of both the magnitude and peak position of the photoresponsivity spectra as the gate voltage changes. This heating effect is further confirmed by the temperature and laser-power-dependent characterization of the photoresponse. In addition, we investigate the spatially-resolved photocurrent, finding that the superconductivity is inhomogeneous across the junction area. Our results provide a new platform for designing tunable superconducting photodetector and indicate that the photoresponse could be a powerful tool in studying the local electronic properties and phase transitions in low-dimensional superconducting systems.

## 1. Introduction

As a two-dimensional (2D) transition metal dichalcogenide with a strong electronic correlation, niobium diselenide (NbSe_2_) has gathered tremendous attention due to its unique collective electronic properties [1,2,3,4]. Quantum transport measurements and scanning tunneling microscopy (STM) show that the atomically thin NbSe_2_ is a hole metal [5], which successively undergoes a charge density wave (CDW) and a superconducting phase transition as the temperature decreases [6,7,8,9,10]. By forming a NbSe_2_-based van der Waals (vdWs) heterostructure, the superconducting order can interact with other electronic phases and elementary excitations at the interface [11], generating abundant novel quantum phenomena, such as the Majorana zero modes [12,13,14], the segmented Fermi surface [15], the proximity induced superconductivity [12,13,14,15,16,17,18,19,20,21,22], and the Andreev reflection [12,23,24,25].

Recently, the photoresponse of the 2D NbSe_2_ has been extensively studied near its superconducting phase transition temperature *T*_c_ [26,27,28]. The photocurrent is generated by the light-induced hot electrons or quasiparticles, which leads to a resistance change of the voltage-biased NbSe_2_ due to the disruption of the superconductivity (for *T* < *T*_c_) or the photo-bolometric effect (for *T* > *T*_c_) [26]. The photoresponse of the NbSe_2_ points to a new means to probe the properties of its charge carriers out of thermal equilibrium and is promising for achieving ultrasensitive and broadband photodetection [26,29]. However, due to the high carrier density, the photocurrent in NbSe_2_ and the resistance of the device can be hardly tuned electrostatically by applying a gate voltage [30,31], limiting its potential application as a superconducting nanowire single-photon detector (SNSPD), in which an adjustable resistor connected in series may increase the device’s speed and avoid latching problems [32]. In addition, many unique quantum phenomena such as the specular interband Andreev reflection in a superconductor-metal/semimetal interface occur only when the Fermi level is kept within a certain range. The lack of gate tunability strongly restricts the observation of such quantum effects.

Here, we utilize a NbSe_2_-graphene lateral heterostructure to realize the gate-controllable 2D superconducting photodetector. Graphene is a Dirac semimetal with highly tunable electrical properties [33]. Its weak electron-acoustic phonon coupling and high intrinsic mobility enable efficient hot carrier generation and transportation [34,35,36,37,38]. In the experiment, we pass a DC through the NbSe_2_-graphene heterostructure to create hot carriers in graphene by Joule heating, which are injected into the NbSe_2_ and modulate its superconductivity. We then systemically carry out the photocurrent measurements by irradiating the NbSe_2_ region with a focused laser beam and observe a gate-dependent photoresponsivity, which is dramatically suppressed when the Fermi level of the monolayer graphene is adjusted to the charge neutrality point. The gate-tunable photoresponse, which is also sensitive to the bias voltage and the temperature, can be well explained by the hot electron effect [39] and is consistent with the electrical transport characterization of the device. Therefore, our device provides a new platform for realizing heterogeneous integration of tunable impedance with high quality in a superconducting photodetector. This simple but useful heterostructure may not only help to optimize the *L*/*R* time constant and increases the speed of the device, but also enables direct modulation of the Joule heat to avoid surpassing the cooling ability which causes latching to occur. Moreover, our spatially resolved photocurrent measurement indicates the presence of nonuniform superconductivity in the device. This observation implies that our technique is suitable for probing the behavior of charge carriers and collective modes locally in a superconducting system, such as proximity-induced superconductivity.

## 2. Methodology

### 2.1. Device Fabrication

The NbSe_2_-graphene heterojunction is fabricated on a 285 nm SiO_2_/Si substrate following the standard electron-beam lithography and vdWs dry transfer procedures: The NbSe_2_ nano-flake was mechanically exfoliated in a glovebox filled with nitrogen gas and transferred onto the pre-patterned Ti/Au (5 nm/35 nm) electrodes using a polypropylene carbonate(PPC)/polydimethylsiloxane (PDMS) stamp [40]. Next, the few-layer graphite (FLG), hexagonal boron nitride (hBN), and the monolayer graphene (MLG) were cleaved from their bulk crystals and stacked together with the NbSe_2_ using a polycarbonate(PC)/polydimethylsiloxane(PDMS) stamp to form the gate-tunable heterojunction. The fabricated device was heated at *T* = 200 °C for 2 h and a 10-min current annealing with *I*_anneal_ = 2 mA [23] was then applied through the device to decrease the contact resistance and improve the photodetector’s performance.

### 2.2. Electrical Transport Measurements

The low-temperature electrical transport measurements were carried out in a closed-cycle optical cryostat (Montana Instruments Corp.; S50) with a base temperature of 3.5 K. The ac and dc bias voltage/current were applied by a digital lock-in amplifier (Stanford Research; SR830) and a multifunction I/O device (National Instruments; USB-6363), respectively. For the differential resistance measurements, a constant ac bias current of 500 nA at 13.371 Hz was passed through the device and the ac electrical potential difference between the voltage probes was recorded as a function of the scanned dc bias current/voltage. The temperature-dependent resistance measurement was performed using a similar geometry to the differential resistance characterization.

### 2.3. Photoresponse Measurements

The photoresponse measurements were carried out using the same optical cryostat set-up as the electrical transport measurements. The dc bias voltage/current and the gate voltage were applied to the device using a multifunction I/O device (National Instruments; USB-6363). The power of the focused continuous-wave laser (*λ* = 532 nm, spot size ~1.5 μm) was modulated by a mechanical chopper at *f* = 183 Hz. The ac photocurrent was measured by a current preamplifier (DL Instruments; Model 1211) and a lock-in amplifier (Stanford Research; SR830). For the scanning photocurrent microscopy (SPCM), the laser beam was raster-scanned by a customized two-axis piezo nanopositioner (Nano Motions Technology CO., Ltd., Shanghai, China) while the real-time position-dependent photocurrent was recorded.

## 3. Results and Discussion

**Figure 1:**
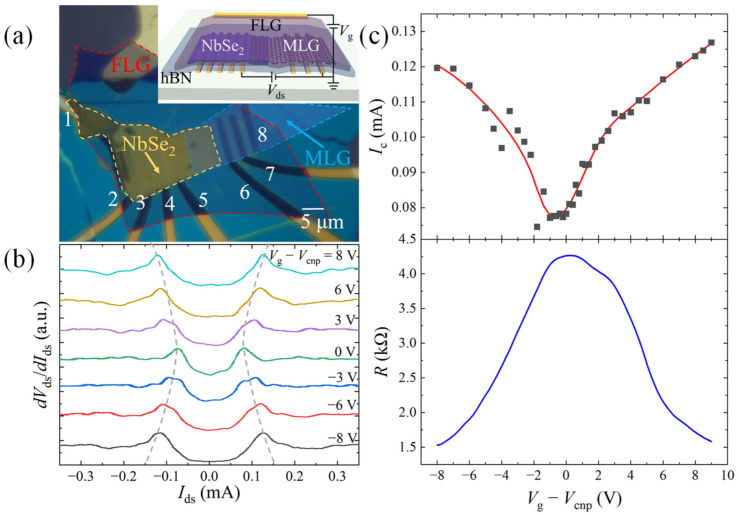
Device structure and the electrical transport characterization of a NbSe_2_-graphene heterojunction. (**a**) Optical microscope image of a multi-terminal device with false coloring for clarity. The edge of the NbSe_2_, MLG, and FLG nano-flakes is marked by the yellow, blue, and red dashed line, respectively. The metal electrodes are numbered from 1 to 8. Inset: Schematic of the NbSe_2_-graphene heterojunction device. (**b**) Differential resistance *dV*_ds_/*dI*_ds_ as a function of the bias current *I*_ds_ at selected top gate voltages *V*_g_ − *V*_cnp_. The dc bias current flows from electrodes 3 to 8, while the voltage is measured between electrodes 4 and 5. (**c**) Top: The critical current *I*_c_ as a function of the top gate voltage *V*_g_ − *V*_cnp_ (black square dots) extracted from (**b**). The red solid line is added as a guide to the eye. Bottom: Gate-dependent two-probe (electrodes 6 and 8) resistance of the MLG.

As introduced in the previous section, the device is fabricated by stacking the exfoliated MLG and the NbSe_2_ nano-flake to form a heterojunction on the pre-patterned metal electrodes using the dry-transfer technique. The whole junction area is coved by an FLG/hBN heterostructure, which serves as the top gate. The optical microscope image and the device schematic of a typical NbSe_2_-graphene field effect transistor with a junction area of about 6 × 10 μm^2^ are shown in Figure 1a. The thickness and the *T*_c_ of the NbSe_2_, determined by the atomic force microscopy (AFM) and the four-probe transport characterization, are 45 nm and 5.14 K by BCS fitting [7] in this device (See Figures S1 and S2). Firstly, we carry out the differential resistance *dV*_ds_/*dI*_ds_ measurement at *T* = 3.9 K to probe the gate-dependent superconductivity of the NbSe_2_-MLG junction. As shown in Figure 1b, two symmetric peaks, denoting the superconducting phase transition critical current *I*_c_ of the NbSe_2_, are observed in each *dV*_ds_/*dI*_ds_ spectrum, which displays remarkable shift with the applied gate voltage *V*_g_ − *V*_cnp_ (*V*_cnp_ denotes to the gate bias at the charge neutrality point of the MLG). In Figure 1c, we plot *I*_c_, which is extracted from the peak position in Figure 1b, as a function of *V*_g_ − *V*_cnp_ and compare it with the gate-dependent resistance of the MLG. It is clearly observed that *I*_c_ is reduced with decreased doping of the MLG and reaches a minimum value of 74 μA near its charge neutrality point. This phenomenon can be explained by the Joule heating effect [39] in the circuit: For fixed *I*_ds_, more hot electrons are generated in graphene when its Fermi level is tuned close to the charge neutrality, resulting in a temperature increase of the charge carriers in the device and thus leading to a diminished *I*_c_.

**Figure 2:**
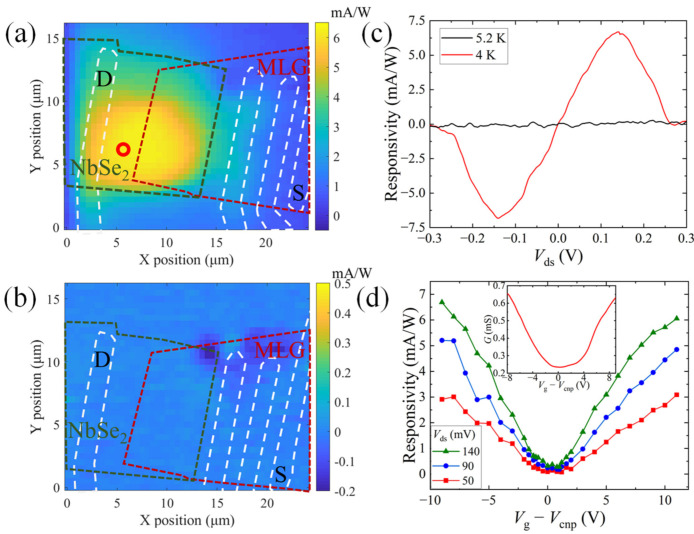
Photoresponse of the NbSe_2_-graphene heterojunction. (**a**,**b**) Spatial photoresponsivity map at *T* = 4.0 K (**a**) and 10.0 K (**b**). A dc bias voltage of *V*_ds_ = 140 mV is applied between the S and D electrodes and *V*_g_ − *V*_cnp_ = −9 V. The green/red/white dashed lines show the geometry of the NbSe_2_, MLG and the electrodes, respectively. The red circle in (**a**) corresponds to the position of the laser spot for the measurement in (**c**,**d**). (**c**) Photoresponsivity as a function of the bias voltage *V*_ds_ at *V*_g_ − *V*_cnp_ = −9 V above (black line) and below (red line) the superconducting transition temperature of the NbSe_2_. (**d**) Photoresponsivity as a function of the gate voltage *V*_g_ − *V*_cnp_ at selected bias voltages. The laser power is fixed at *P* = 20 μW for (**a**–**d**). Inset: Gate-dependent two-probe conductance of the MLG in the dark.

We next investigate the photoresponsivity (defined as the photocurrent per unit incident laser power) of our NbSe_2_-MLG device at low temperatures using the SPCM technique: A constant DC bias voltage of *V*_ds_ = 140 mV is applied through the junction and the top-gate voltage is fixed at *V*_g_ − *V*_cnp_ = −9 V, while the photocurrent *I*_ph_ is measured by raster-scanning the focused laser beam (wavelength *λ* = 532 nm, laser power *P* = 20 μW) across the device. As displayed in Figure 2a, notable photoresponse is observed on the NbSe_2_ side of the junction at *T* = 4.0 K, which gradually decreases with the laser spot moving across the junction to the MLG side. The photoresponse disappears when the temperature is higher than *T*_c_ (Figure 2b). More results with different applied bias voltages can be seen in Figure S3. We then fix the laser beam on NbSe_2_ and plot the photoresponsivity as a function of *V*_ds_ (Figure 2c). The fact that responsivity shows an anti-symmetrical dual-peaked feature for *T* < *T*_c_ [26] and is reduced by at least two orders of magnitude for *T* > *T*_c_ confirms that the photoresponse originates from the photo-induced superconducting phase transition in NbSe_2_. We next show that the photoresponsivity is gate-tunable by measuring *I*_ph_ versus *V*_g_ − *V*_cnp_ in Figure 2d. At all selected bias voltages, an ambipolar photoresponsivity signal is observed, which is minimized near the charge neutrality point of the MLG. The ambipolar gate-modulation of the photoresponsivity is similar to the gate-dependent conductance of the MLG, implying that the magnitude of responsivity is directly related to the position of the graphene’s Fermi level.

**Figure 3:**
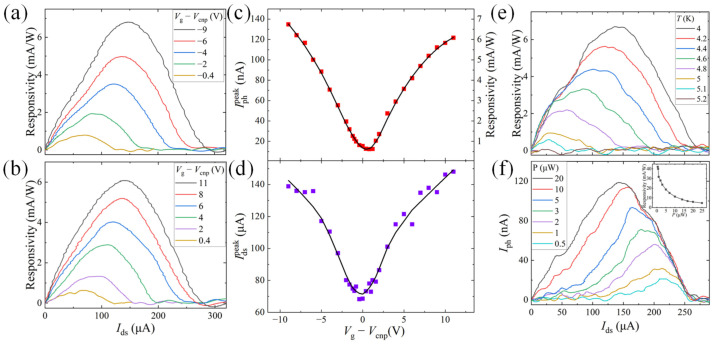
Modulation of the photoresponse in the superconducting NbSe_2_-MLG heterostructure. (**a**,**b**) Photoresponsivity as a function of the bias current at selected top gate voltages for *p*-doped (**a**) and *n*-doped graphene (**b**), respectively. The laser power is *P* = 20 μW and the temperature is *T* = 4.0 K. (**c**,**d**) The maximum photocurrent $I_{ph}^{peak}$ (red square dots in (**c**)) and the peak position $I_{ds}^{peak}$ (purple square dots in (**d**)) extracted from (**a**,**b**) as a function of the top gate voltages, respectively. The black solid lines are added as a guide to the eye. (**e**) Photoresponsivity versus *I*_ds_ at selected temperatures. The gate voltage and laser power are kept at *V*_g_ − *V*_cnp_ = −9 V and *P* = 20 μW. (**f**) Photocurrent *I*_ph_ versus *I*_ds_ at selected laser powers with *V*_g_ − *V*_cnp_ = −9 V and *T* = 4.0 K. Inset: The maximum photoresponsivity (black square dots) extracted from (**f**) as a function of the laser power.

To further analyze the gate dependence of the photoresponse, we measure the photoresponsivity spectra (responsivity as a function of the bias current *I*_ds_) at selected *V*_g_ − *V*_cnp_ for both *p*- (Figure 3a) and *n*-doped (Figure 3b) graphene. In each spectrum, responsivity shows a broad peak, which is enhanced and shifts to right with increasing carrier density of the MLG. We extract the magnitude ($I_{ph}^{peak}$) and the position ($I_{ds}^{peak}$) of each peak from the measured photoresponsivity spectra and plot them as a function of *V*_g_ − *V*_cnp_ in Figure 3c,d, respectively. The fact that the gate dependence of $I_{ph}^{peak}$ and $I_{ds}^{peak}$ is similar to that of the NbSe_2_′s differential resistance in Figure 1c, suggests that they share the same mechanism: Ignoring the contact resistance, the Joule heating power (*P* = *I*_ds_^2^*R*) of the device for a fixed bias current *I*_ds_ is proportional to the resistance *R* of the MLG, which is tunable by the top gate voltage. The carrier temperature can therefore increase significantly, when the MLG is gated to the charge neutrality point, leading to a decreased superconducting critical current *I*_c_ of the NbSe_2_. Correspondingly, the photoresponsivity peak, determined by the photo-induced superconducting phase transition, shifts to left in the spectrum with reduced magnitude. To verify this hypothesis, we directly measure the temperature-dependent photoresponsivity spectra for a fixed *V*_g_ − *V*_cnp_ = −9 V and the excitation power of *P* = 20 μW. As shown in Figure 3e, as the temperature increases, the photoresponsivity is attenuated with its peak position shifting to lower *I*_ds_, qualitatively consistent with the gate modulation to responsivity in Figure 3a,b. We further investigate the power dependence of the photoresponse at *T* = 4.0 K (Figure 3f): As the power changes from 0.5 μW to 20 μW, the size of the hotspot generated via the optical energy injection in NbSe_2_ gradually becomes larger, resulting in an enhanced photocurrent signal. At the same time, the peak position of *I*_ph_ shifts to the left due to the photo-induced temperature rise. Moreover, the photoresponsivity increases quickly with reduced laser power and reaches a maximum value of 42 mA/W at 0.5 μW, suggesting that our photodetector is advantageous in probing weak electromagnetic radiations. The characterization of the photoresponsivity and photocurrent spectra at different *T* and *P* (also see Figures S4 and S5) further confirms that the thermal effect plays an important role in tuning the photoresponse with the top gate voltage. We notice that the photoresponsivity and photocurrent signal is slightly different from each other in Figure 3a,e,f even for the same experimental conditions, which is possibly due to the laser beam readjustment and the degradation of the device after repeated electrical scans.

Table 1 lists the performance of several photodetectors based on conventional and low-dimensional superconductors. In the table, our NbSe_2_-MLG device shows acceptable responsivity. Its response time is estimated to be at the order of 0.5 ns and the detection wavelength can be extended to the infrared range. Compared with other superconducting photodetectors, our gate-tunable NbSe_2_-MLG heterojunction offers additional degrees of freedom, which makes it controllable and may further enhance the response speed. Considering that the responsivity of the device is restricted by the relatively large resistance of MLG, we expect the performance of the device could be further enhanced by improving the mobility of graphene or replacing it with other gate-tunable semimetals with lower resistance and higher mobility.

**Figure 4:**
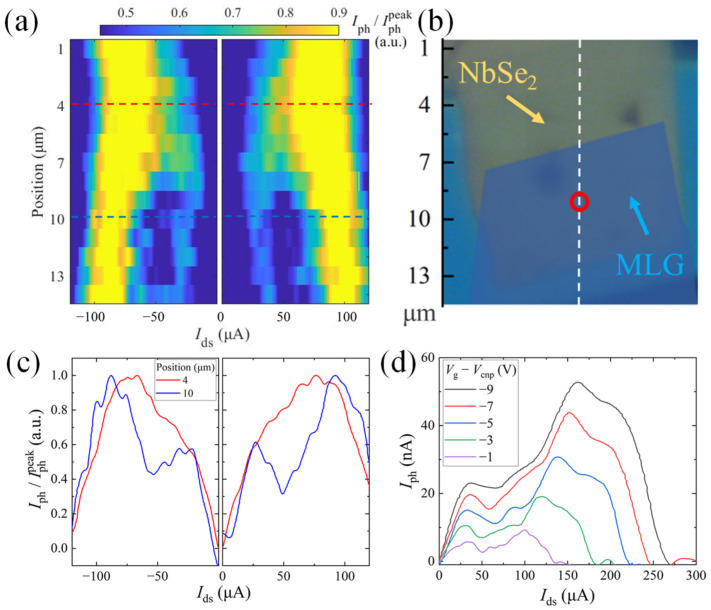
Laser spot position dependence of the photocurrent. (**a**) Spatially-resolved normalized photocurrent spectra of the NbSe_2_-MLG heterojunction. The laser beam is scanned across the white dashed line in (**b**) with the step size of 1 μm. Each spectrum is divided by the peak (for positive bias current)/dip (for negative bias current) photocurrent to become normalized. (**b**) Optical microscope image of the device. The red circle corresponds to the position of the laser spot for the measurement in (**d**). (**c**) Normalized photocurrent *I*_ph_/$I_{ph}^{peak}$ versus *I*_ds_ at selected laser beam positions marked by the red and blue dashed lines in (**a**). (**d**) Photocurrent *I*_ph_ versus *I*_ds_ at selected gate voltages *V*_g_ − *V*_cnp_ in the junction area. For all measurements in (**a**–**d**), the laser power and gate voltage are fixed at *P* = 10 μW and *V*_g_ − *V*_cnp_ = −1 V, *T* = 4.0 K.

Finally, we point out that our technique could be utilized to locally probe the superconductivity and electronic phase transitions. To demonstrate this, the laser spot is scanned across the NbSe_2_-MLG heterojunction (along the white dashed line shown in Figure 4b), while the photocurrent is measured as a function of *I*_ds_. In Figure 4a, we show the normalized photocurrent spectra for different beam positions using a 2D plot. When the focused laser is parked at the bare NbSe_2_, a single broad peak shows up, which is split into two parts as the beam moves to the junction area (Figure 4c). This observed double-peak feature is further investigated by checking its gate dependence (Figure 4d). The peak at higher bias current shifts to the left with the gate voltage tuning close to graphene’s charge neutrality point, which we attribute to the photoresponse induced by the superconducting phase transition in NbSe_2_. The other peak appears at a lower bias current, which barely shifts with *V*_g_. This second peak might be relevant to some inhomogeneity of the electronic phase in the junction area or the proximity-induced superconductivity in MLG. More experiments are still necessary to clarify the underlying physical mechanism.

## 4. Conclusions

In summary, the gate-modulated photoresponse in a NbSe_2_-graphene superconducting heterostructure is systematically explored. Hot carriers generated by the electrical Joule heating, which is directly associated with the gate-dependent resistance of the MLG, can effectively tune the photoresponsivity signal. Moreover, the spatially resolved photocurrent measurement is confirmed to be capable of probing the nonuniformity of the superconductivity and related phase transitions. Our result thus provides a model system for studying the behavior of 2D correlated electrons and is potentially useful for making new kinds of vdW optoelectronic devices.

## Figures and Tables

**Table 1:** Comparison between photodetectors based on superconductors.

Materials	Responsivity (A/W)	Response Time *τ*	Wavelength *λ*	Temperature *T*	Gate Tunability	Ref.
NbSe_2_-MLG	42 m	~0.5 ns (In theory)	532 nm (IR applicable)	4.0 K	√	This work
NbN	~ 0.16	45 ps	395 nm	2.15 K	×	[41]
NbSe_2_	~ 3	2.4 ns	1550 nm	5 K	×	[28]
NbSe_2_	43.2	~0.5 ns (In theory)	727 nm (IR applicable)	3.8 K	×	[26]

## Data Availability

The data that support the findings of this study are available within the article and its Supplementary Materials. Further data are available from the corresponding author upon reasonable request.

## Supplementary Materials

The following supporting information can be downloaded at: https://www.mdpi.com/article/10.3390/nano13030421/s1, Figure S1: Sample thickness characterization. (a) Optical micrograph of the device replotted from Figure 1a in the main text. (b,c) AFM image of the NbSe_2_ (b) and hBN (c) nano-flake, respectively; Figure S2: Electrical transport characterization of the NbSe_2_. (a) Four-probe resistance of the NbSe_2_ as a function of the temperature. (b) Differential resistance *dV*_ds_/*dI*_ds_ as a function of the bias current *I*_ds_ at selected temperatures. (c) The critical current *I*_c_ extracted from (b) as a function of the temperature *T* (red square dots). The black solid line shows the BCS fit of the measured ∆E versus *T*; Figure S3: Spatial photoresponsivity maps of the NbSe_2_-MLG heterojunction. (a–d) Spatial photoresponsivity maps at different temperatures and bias voltages: *T* = 4.0 K, *V*_ds_ = 0 mV (a), *T* = 4.0 K, *V*_ds_ = 50 mV (b), *T* = 10.0 K, *V*_ds_ = 0 mV (c), *T* = 10.0 K, *V*_ds_ = 50 mV (d); Figure S4: Temperature dependence of the gate-tunable photoresponse. (a–f) Photoresponsivity as a function of the bias current *I*_ds_ at selected temperatures for *V*_g_ − *V*_cnp_ = −9 V (a), −7 V (b), −5 V (c), −3 V (d), −1 V (e), and 1 V (f); Figure S5: Power dependence of the gate-tunable photoresponse. (a–c) Photocurrent *I*_ph_ as a function of the bias current *I*_ds_ at selected excitation powers for *V*_g_ − *V*_cnp_ = −5 V (a), −1 V (b) and 600 mV (c). (d) Photoresponsivity extracted and calculated from the spectra in (a–c) and Figure 3f in the main text as a function of the laser power at selected gate voltages. All measurements are carried out at *T* = 4.0 K; Figure S6: Gate dependence of the device’s resistance. Four-probe resistance *R* of the NbSe_2_-MLG junction as a function of the bias current *I*_ds_ at selected gate voltages. The calculated *R* is derived by integrating the differential resistance spectra in Figure 1b in the main text.
