# Supplementary material for: Tunable Photoresponse in a Two-Dimensional Superconducting Heterostructure
Source: Nanomaterials (Basel). 2023 Jan 19;13(3):421. doi: 10.3390/nano13030421 (PMC9920438; doi:10.3390/nano13030421)
Supplement: Supplementary file 1 [file nanomaterials-13-00421-s001.zip › nanomaterials-2086426-supplementary.pdf]

nanomaterials

Supplementary Materials

Tunable Photoresponse in a Two-Dimensional Superconducting Heterostructure

Zijie Ji [1,2], Ruan Zhang [1,2], Shuangxing Zhu [1,2], Feifan Gu [1,2], Yunmin Jin [1,2], Binghe Xie [1,2], Jiaxin Wu [1,2] and Xinghan Cai [1,*]

[1] National Key Laboratory of Science and Technology on Micro/Nano Fabrication, Shanghai Jiao Tong University, Shanghai 200240, China; jizijie@sjtu.edu.cn (Z.J.); zhangruan@sjtu.edu.cn (R.Z.); zsx_sjtu2021@sjtu.edu.cn (S.Z.); gufeifan@sjtu.edu.cn (F.G.); jym15122817@sjtu.edu.cn (Y.J.); binghe.xie@sjtu.edu.cn (B.X.); wujiaxin@sjtu.edu.cn (J.W.)
[2] Department of Micro/Nano Electronics, School of Electronic Information and Electrical Engineering, Shanghai Jiao Tong University, Shanghai 200240, China
* Correspondence: xhcai@sjtu.edu.cn

Supplementary note 1. Atomic force micrographs of the hexagonal boron nitride and $NbSe_2$.

The thickness of the exfoliated 2D materials is determined by the atomic force microscopy (AFM). For the device we discussed in the main text, the thickness of the $NbSe_2$ and hBN nano-flakes are 45 nm and 20 nm, respectively (Figure. S1b and S1c). To avoid sample degradation, the AFM image of the $NbSe_2$ is taken after it has been covered by the top hBN.

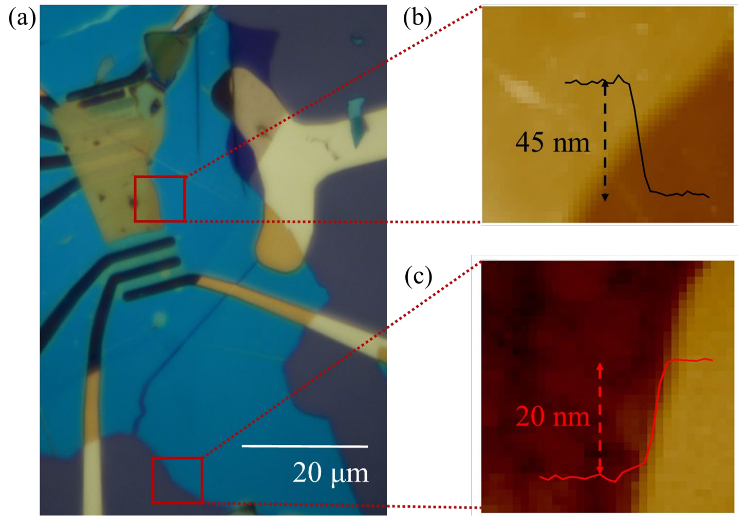

Figure S1. Sample thickness characterization. (**a**) Optical micrograph of the device replotted from Figure 1a in the main text. (**b, c**) AFM image of the $NbSe_2$ (**b**) and hBN (**c**) nano-flake, respectively.

Supplementary note 2. Low temperature transport characterization of the $NbSe_2$.

The superconductivity of the $NbSe_2$ nano-flake used in the main text is characterized by the four-probe electrical transport measurements. Figure S2a shows its resistance $R - R_{residual}$ as a function of the temperature by passing a constant current through the electrode 1 and 5 and measuring the voltage drop between the electrode 2 and 4 (see Figure 1a in the main text). A rapid decrease of $R - R_{residual}$ occurs between 5 and 6 K, indicating the superconducting phase transition of the $NbSe_2$. To further characterize the superconductivity, the differential resistance dV_{ds}/dI_{ds} as a function of the bias current at selected temperatures are plotted in Figure S2b. At $T = 4.2$ K, two remarkable symmetric peaks are ob-

served at the superconducting critical current $I_c = \pm$ 0.6 μA. These BCS peaks are broadened and gradually shift to lower bias current with increasing temperature and eventually disappear above 4.9 K. We extract the superconducting gap ΔE at different temperatures from the differential resistance spectra and fit to BCS theory using $\Delta E \propto \tanh(1.74\sqrt{\frac{T_c}{T}-1})$[1], which gives a superconducting critical temperature of T_c = 5.14 K as illustrated in Figure S2c.

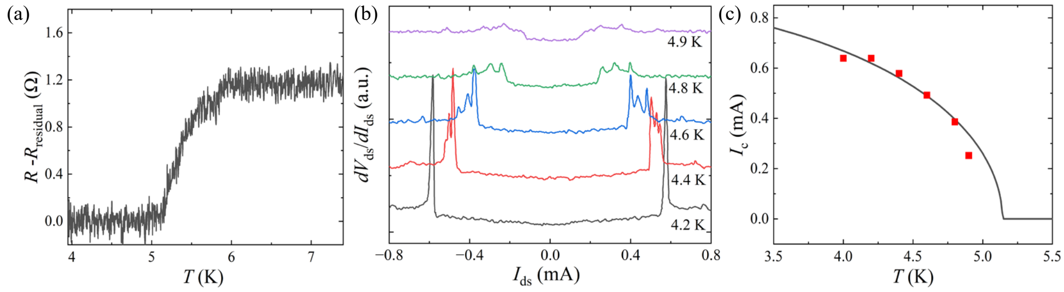

Figure S2. Electrical transport characterization of the $NbSe_2$. (**a**) Four-probe resistance of the $NbSe_2$ as a function of the temperature. (**b**) Differential resistance dV_{ds}/dI_{ds} as a function of the bias current I_{ds} at selected temperatures. (**c**) The critical current I_c extracted from (**b**) as a function of the temperature T (red square dots). The black solid line shows the BCS fit of the measured ΔE versus T.

Supplementary note 3. SPCM images of the $NbSe_2$-graphene heterojunction.

The SPCM images with different applied bias voltages V_{ds} are taken below (Figures S3a–S3b) and above (Figures S3c–S3d) the critical temperature T_c, respectively. No noticeable photoresponse around the junction area has been observed at V_{ds} = 0 mV or at $T > T_c$ (Figure S3a, S3c–d). Below T_c, large photoresponsivity shows up at the $NbSe_2$ side of the junction when a bias voltage of V_{ds} = 50 mV is applied (Figure S3b). Comparing with Figure 2 in the main text, the photoresponsivity further increases with V_{ds} and is peaked near the superconducting critical voltage. These observations confirm that the photoresponse is induced by the superconducting phase transition occurring in the device.

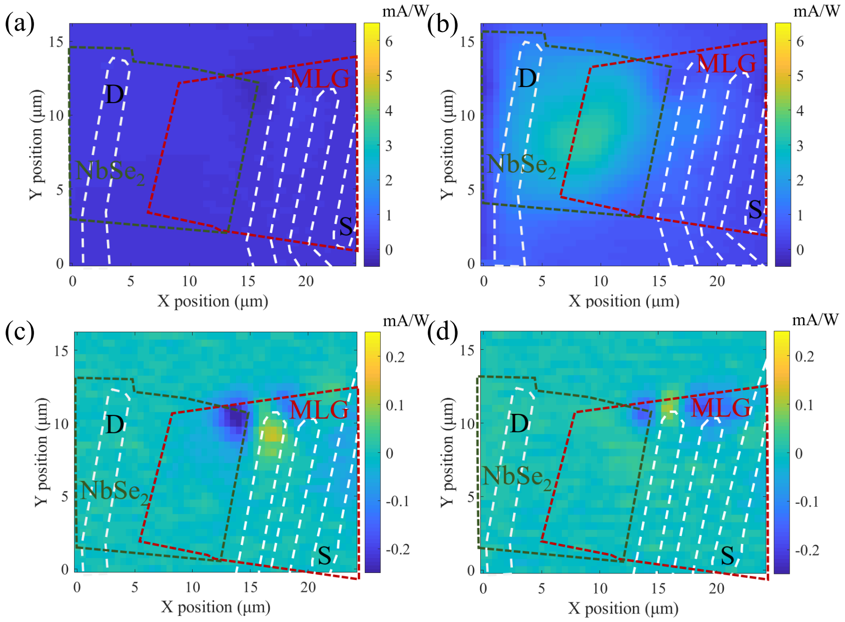

Figure S3. Spatial photoresponsivity maps of the $NbSe_2$-MLG heterojunction. (**a**–**d**) Spatial photoresponsivity maps at different temperatures and bias voltages: T = 4.0 K, V_{ds} = 0 mV (**a**), T = 4.0 K, V_{ds} = 50 mV (**b**), T = 10.0 K, V_{ds} = 0 mV (**c**), T = 10.0 K, V_{ds} = 50 mV (**d**).

Supplementary note 4. Temperature dependence of the gate-tunable photoresponse in the $NbSe_2$-graphene heterojunction.

Figure 3e in the main text shows the photoresponsivity spectra taken at varying temperatures up to 5.2 K with a fixed gate voltage of $V_g - V_{cnp} = -9$ V. The same measurements are repeated here by tuning the Fermi level of the MLG from the heavily *p*-doped regime (Figure S4a, replotted from Figure 3e) to low-doped regime (Figure S4f). Although both the peak photoresponsivity and the critical current become smaller with decreasing carrier density of the MLG, the photoresponsivity spectrum shows similar temperature dependence at different gate voltages, consistent with our discussion in the main text. Interestingly, we point out that despite of the strongly suppressed photoresponse near the graphene's Dirac point, the critical temperature T_c (i.e. the temperature at which the photoresponsivity disappears) stays unchanged ($T_c \sim 5.2$ K), indicating that the gate modulation to the photoresponse is induced by the Joule heating effect when a bias current is applied. The lattice temperature is not affected by simply tuning the electrostatic doping.

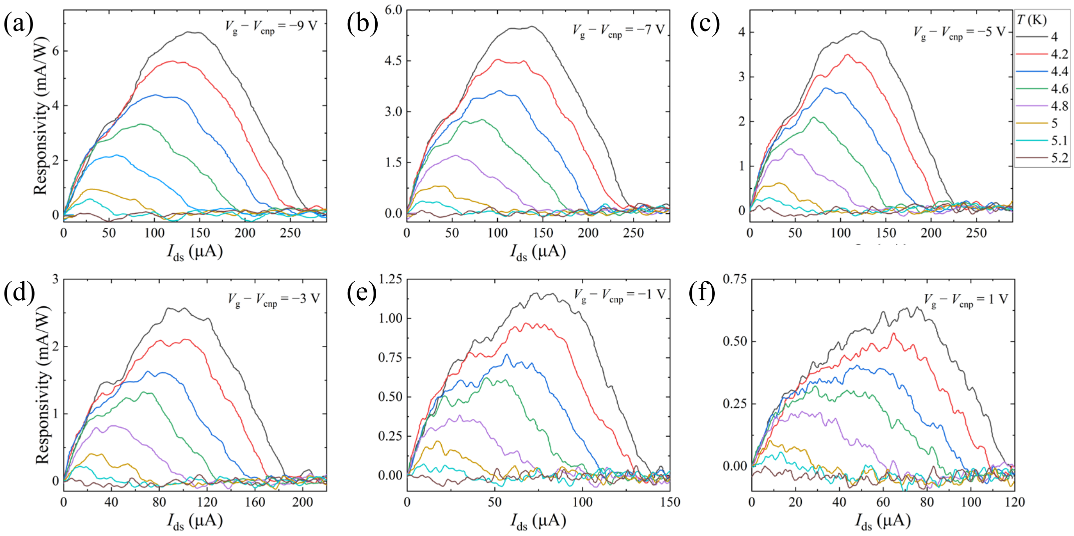

Figure S4. Temperature dependence of the gate-tunable photoresponse. (**a**–**f**) Photoresponsivity as a function of the bias current I_{ds} at selected temperatures for $V_g - V_{cnp} = -9$ V (**a**), −7 V (**b**), −5 V (**c**), −3 V (**d**), −1 V (**e**), and 1 V (**f**).

Supplementary note 5. Power dependence of the gate-tunable photoresponse in the $NbSe_2$-graphene heterojunction.

In the main text, the photocurrent spectra at selected laser powers with an applied gate voltage of $V_g - V_{cnp} = -9$ V are displayed in Figure 3f. Here, we show the power-dependent photocurrent spectra at different V_g in Figure S5a–S5c to further explore the laser power modulation to the photoresponse. As the graphene's Fermi level is gradually tuned to the charge neutrality point, the photocurrent is reduced with its peak position shifting to lower bias current. The photoresponsivity calculated from the peak photocurrent of each spectrum extracted from Figure S5a–S5c and Figure 3f in the main text are plotted as a function of the excitation power in Figure S5d. At all selected gate voltages, the photoresponsivity increases quickly with reduced laser power, suggesting that our photodetector is advantageous in probing weak electromagnetic radiations.

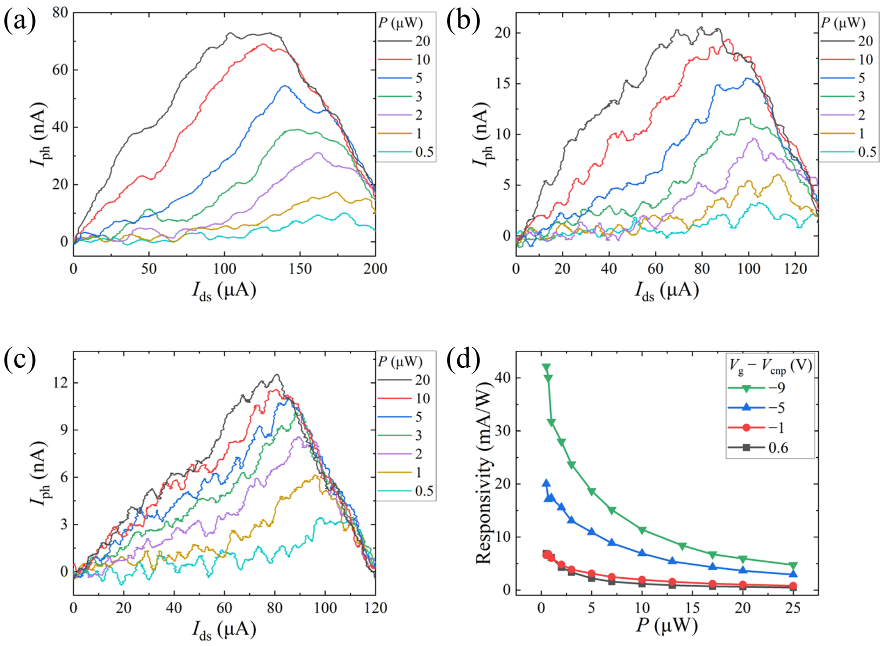

Figure S5. Power dependence of the gate-tunable photoresponse. (**a–c**) Photocurrent I_{ph} as a function of the bias current I_{ds} at selected excitation powers for $V_g - V_{cnp} = -5$ V (**a**), −1 V (**b**) and 600 mV (**c**). (**d**) Photoresponsivity extracted and calculated from the spectra in (**a–c**) and Figure 3f in the main text as a function of the laser power at selected gate voltages. All measurements are carried out at $T = 4.0$ K.

Supplementary note 6. Gate dependence of the four-probe resistance of the $NbSe_2$-MLG junction.

In the main text, we characterized the differential resistance dV_{ds}/dI_{ds} as a function of the bias current at selected gate voltages $V_g - V_{cnp}$ in Figure 1b. Here, to further illustrate the relationship between the bias current I_{ds} and the superconducting phase transition, the four-probe resistance R *vs* the bias current I_{ds} is derived by doing the integral of the dV_{ds}/dI_{ds} - I_{ds} spectrum. As shown in Figure S6, two symmetric resistance steps are observed in each R - I_{ds} curve, denoting to the superconducting phase transition. As the graphene's Fermi level is tuned to the charge neutrality point, the resistance steps gradually shift to lower bias current.

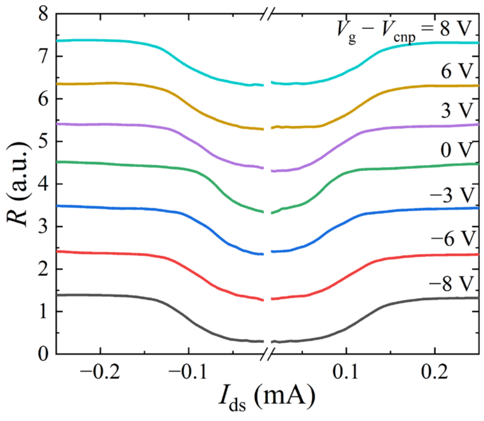

Figure S6. Gate dependence of the device's resistance. Four-probe resistance R of the $NbSe_2$-MLG junction as a function of the bias current I_{ds} at selected gate voltages. The calculated R is derived by integrating the differential resistance spectra in Figure 1b in the main text.

References

1. Khestanova, E.; Birkbeck, J.; Zhu, M.; Cao, Y.; Yu, G.L.; Ghazaryan, D.; Yin, J.; Berger, H.; Forro, L.; Taniguchi, T.; et al. Unusual Suppression of the Superconducting Energy Gap and Critical Temperature in Atomically Thin $NbSe_2$. *Nano Lett.* **2018**, *18*, 2623-2629.
